# The Role of Different Feedback Devices in the Survival of Patients in Cardiac Arrest: Systematic Review with Meta-Analysis

**DOI:** 10.3390/jcm13195989

**Published:** 2024-10-08

**Authors:** Luca Gambolò, Pasquale Di Fronzo, Giuseppe Ristagno, Sofia Biserni, Martina Milazzo, Delia Marta Socaci, Leopoldo Sarli, Giovanna Artioli, Antonio Bonacaro, Giuseppe Stirparo

**Affiliations:** 1 SIMED—Società Italiana di Medicina e Divulgazione Scientifica, 43121 Parma, Italy; 2 Local Health Unit of Piacenza, Ospedale Guglielmo da Saliceto, 29122 Piacenza, Italy; 3 Department of Medicine and Surgery, University of Parma, 43121 Parma, Italy; 4 Dipartimento di Fisiopatologia Medico-Chirurgica e dei Trapianti, Università Degli studi di Milano, 20122 Milano, Italy; 5 Fondazione IRCCS Ca’ Granda Ospedale Maggiore Policlinico, 20090 Milano, Italy

**Keywords:** feedback devices, cardiopulmonary resuscitation, return of spontaneous circulation, OHCA, IHCA

## Abstract

**Background:** Cardiac arrest is a critical condition affecting approximately 1 in every 1000 people in Europe. Feedback devices have been developed to enhance the quality of chest compressions during CPR, but their clinical impact remains uncertain. This study aims to evaluate the effect of feedback devices on key clinical outcomes in adult patients experiencing both out-of-hospital (OHCA) and in-hospital cardiac arrest (IHCA). The primary objective is to assess their impact on the return of spontaneous circulation (ROSC); secondary objectives include the evaluation of neurological status and survival to discharge. **Methods:** A systematic review was conducted following PRISMA guidelines, utilizing databases including PubMed, Scopus, Web of Science, and Embase. Studies published between 2000 and 2023 comparing CPR with and without the use of feedback devices were included. A fixed-effects network meta-analysis was performed for ROSC and survival, while a frequentist meta-analysis was conducted for neurological outcomes. **Results:** Twelve relevant studies met the inclusion criteria. The meta-analysis results showed that the use of audiovisual feedback devices significantly increases the likelihood of ROSC (OR 1.26, 95% CI 1.13–1.41, *p* < 0.0001) and survival (OR 1.52, 95% CI 1.27–1.81, *p* < 0.0001) compared to CPR without feedback. However, the effect of metronomes did not reach statistical significance. **Conclusions:** Feedback devices, particularly audiovisual ones, are associated with improved clinical outcomes in cardiac arrest patients. Their use should be encouraged in both training settings and real-life emergency scenarios to enhance survival rates and ROSC. However, further studies are needed to confirm long-term impacts and to explore the potential benefits of metronomes.

## 1. Introduction

Cardiac arrest is a very relevant pathology, affecting about 1 in every 1000 people in Europe [1,2]. Studies in recent years have focused predominantly on analyzing cardiac arrests in the out-of-hospital setting (OHCA) [3,4], whereas studies on events in the in-hospital setting (IHCA) are less numerous and show very different epidemiology and outcomes from pre-hospital [5,6].

To improve patient survival, advanced cardiovascular life support (ACLS) algorithms [2,7] and the adherence that healthcare professionals follow in their management have been reported to play a relevant role [8]. The algorithm should be promptly started by all healthcare professionals attending a cardiac arrest; in fact, deviations in terms of timing [9] and the clinical algorithm have been shown to play a negative role in patient outcomes [10,11].

Cardiac arrest guidelines have emphasized the importance of the rapid initiation of CPR and proper maintenance of a standard rate [12] to guarantee proper perfusion to the critical organs. Indeed, despite the role of advanced maneuvers such as ACLS, rapid and effective BLS is necessary for the proper management of advanced maneuvers [13,14]. For this purpose, feedback devices [15] have been developed to help healthcare professionals maintain an adequate rhythm and feedback [16,17]. These devices proved effective during simulation [18] and also during the management of cardiac arrests on the ward [15].

Although the devices have shown their effectiveness [19], they are not yet commonly used in hospital settings and emergencies [20] or even in training settings where healthcare workers become familiar with devices for managing cardiac arrest [21].

The study aims to provide an evaluation of the effect of feedback devices on clinical outcomes in the adult human population during both out-of-hospital cardiac arrest (OHCA) and in-hospital cardiac arrest (IHCA). The primary objective of the study is to evaluate their effect on return to spontaneous circulation (ROSC); the secondary objective is to evaluate the effect on neurological status and survival to discharge.

## 2. Materials and Methods

We conducted a systematic review following the PRISMA (Preferred Reporting Items for Systematic Reviews and Meta-Analyses) guidelines [22].

### 2.1. Eligibility Criteria

Studies published between 2000 and 2023 that examined cardiopulmonary resuscitation performed with the use of feedback devices by both healthcare personnel and lay bystanders were included, including both in-hospital and out-of-hospital cardiac arrests. In addition, the studies were to compare two interventions: feedback device-assisted CPR and conventional CPR (without the use of feedback devices). The language was limited to English, French, and Italian. As our research focused on the human population, studies involving dummy simulations and animal studies were excluded. Commentaries, case reports, case series, posters, narrative reviews, systematic reviews, and meta-analyses were excluded.

### 2.2. Search String

The PICO format was used to construct the search string:

Population: patients in cardiac arrest.

Intervention: use of feedback devices for chest compressions during CPR.

Control: CPR without the use of CPR feedback.

Outcomes: ROSC (primary outcome); survival to discharge, neurological status at discharge (secondary outcomes).

The search was conducted on 30 June 2024. Multiple databases were consulted, as recommended by the main practical guidelines for the synthesis of scientific evidence: PubMed, Scopus, Web Of Science, and Embase.

The entire working group defined and shared the search string and can be found in Supplementary Materials in Table S1 (Research strategy).

### 2.3. Selection of Studies

Search strings were entered into the respective databases with publication after 1 January 2000 as the limit. Papers were uploaded to Rayyan (http://rayyan.qcri.org, last access 30 June 2023), a free web and mobile app used by researchers to speed up the initial screening of articles [23]. The software was used at all stages of paper selection, up to the article inclusion stage.

Data selection was performed independently by four authors (SB, DFC, MM, and DMS), and discrepancies were resolved by consulting a fifth (LG) and a sixth (PD) author.

The LG and PD reviewers extracted data independently from the included studies. Discrepancies were resolved through discussion until a consensus was reached.

The main outcome considered in the research was the rate of return to spontaneous circulation (ROSC). Data on neurological status and survival to discharge were also extracted where available.

Publication bias risk assessment was conducted by LG using funnel plots.

### 2.4. Effect Measurement

For the evaluation of the effect of feedback devices on the return to spontaneous circulation, the odds ratio of ROSC between CPR using feedback devices and conventional CPR was used; the odds ratio between the survival rate of patients in cardiac arrest on whom CPR with feedback devices was performed and the odds ratio between favorable and unfavorable neurological outcomes in patients in cardiac arrest who underwent CPR with feedback devices and without was used to assess the effect of feedback devices on neurological outcomes.

### 2.5. Synthesis Method

We conducted a network meta-analysis with a fixed-effects model for ROSC and survival and a frequentist meta-analysis (again with a fixed-effects model) for neurological outcomes.

This type of statistical approach thus allowed us to evaluate a network of evidence in which we have more than two different interventions. In our case, the studies examined present the application of different feedback devices and their absence during CPR. In the fixed-effects model, it is assumed that the effect of interest is constant between the included studies; therefore, fixed weights are used to combine the results.

We also created networks, forest plots, and funnel plot plots for each analysis. Statistical analysis was performed with R 4.2.1 version with ‘meta’ and ‘netmeta’ packages [24].

## 3. Results

**Figure 1:**
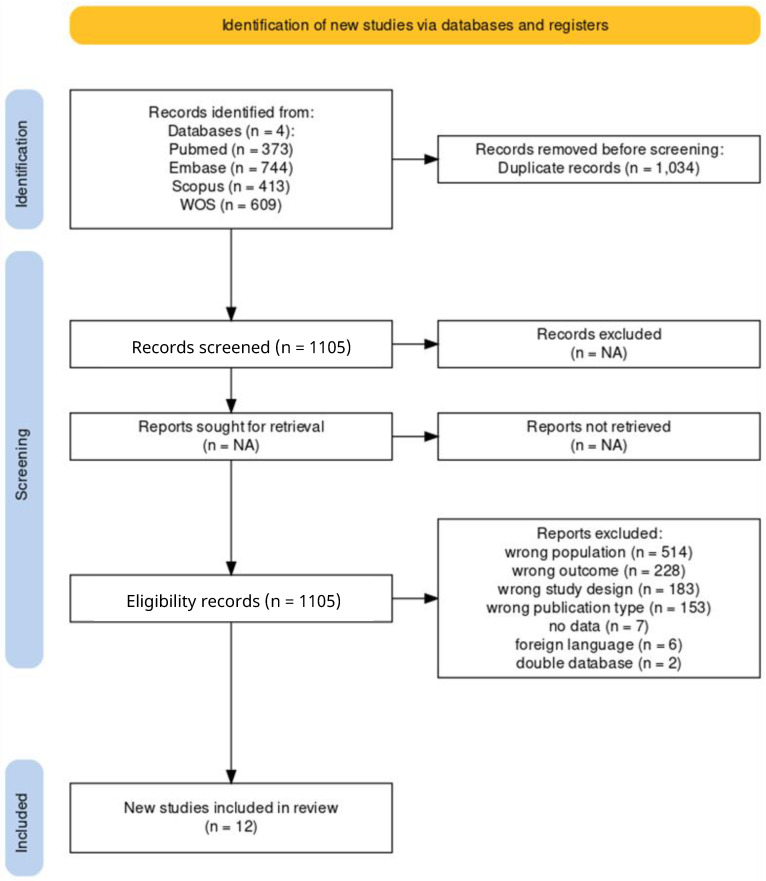
Selection process flow diagram.

The search strings were entered into the respective databases by setting publication after 1 January 2000 as the limit. As shown in Figure 1, the search generated a total of 2139 articles of which 373 were in Pubmed, 744 were in Embase, 413 were in Scopus, and 609 were in Web of Science. All documents were uploaded to Rayyan (http://rayyan.qcri.org) to speed up the initial screening of articles [23]. The software, therefore, allowed the group to perform an initial step of eliminating duplicates, which turned out to be 1034, resulting in a total of 1105 unduplicated articles. Following further screening of articles, 12 studies were included in the meta-analysis [15,16,24,25,26,27,28,29,30,31,32,33]. Figure 1 shows the selection process flow diagram.

Of the 1105 potentially eligible unduplicated studies, 12 met our inclusion criteria. These 12 studies are shown in Table 1.

Table 2 shows the included studies (Study) with the respective outcomes analyzed (Outcome), the type of intervention applied (Feedback), the number of events that achieved the outcome considered (Event), and the total number of events included in the intervention/control group (Total). Several outcomes were analyzed from each study (good neurological status, survival to discharge, and ROSC). For each outcome, an intervention group (feedback devices) and a control group (no feedback devices) were selected.

### 3.1. ROSC

**Figure 2:**
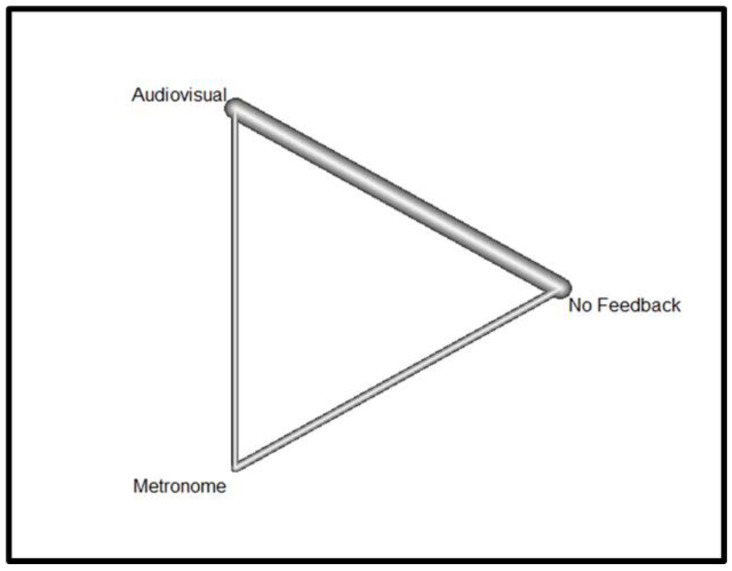
Netgraph For ROSC.

Concerning the ROSC outcome, Figure 2 shows the Netgraph linking direct comparisons of three interventions applied during cardiopulmonary resuscitation in the ROSC studies considered in this meta-analysis: the use of audiovisual feedback devices compared with the use of metronomes, the use of audiovisual feedback devices compared with the absence of feedback devices, and finally the use of metronomes compared with the absence of feedback devices.

The thickness of the line connecting the different interventions represents the overall amount of comparisons between the interventions in the different studies considered; the thicker the line, the greater the number of comparisons between the two interventions. The graph shows that there are more comparisons between the use of audio-visual feedback devices and the non-use of feedback devices during cardiopulmonary resuscitation.

Table 3 summarizes the results of the comparisons between different interventions within their respective studies.

The analysis of the overall effect relative to the applied interventions for ROSC shows OR 1.26 (IC 95% 1.13–1.41, *p* < 0.0001) for audiovisual feedback and an OR of 1.50 (IC 95% 0.98–2.30, *p* = 0.064) for metronome compared to patients managed without feedback.

**Figure 3:**
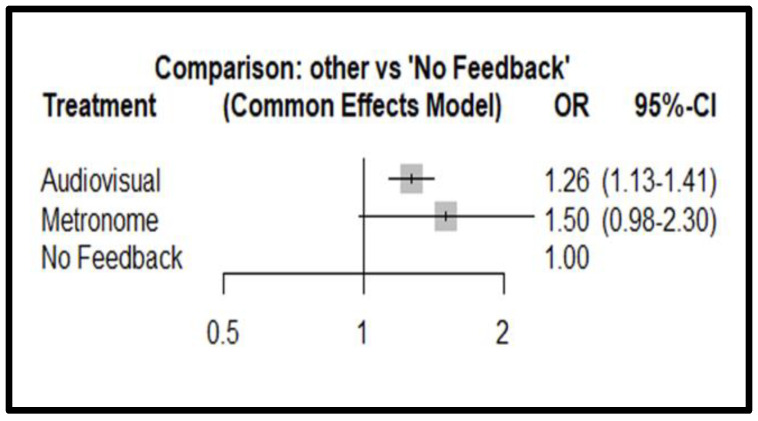
Forest plot for ROSC; Tau = 0.42, Tau^2^ = 0.17, I^2^ = 80.3% (66.4–88.4%).

Figure 3 shows the forest plot for ROSC, while the funnel plot with the distribution of effects versus standard error can be found in Supplementary Materials in Figure S1, Funnel plot for ROSC. 

### 3.2. Survival

**Figure 4:**
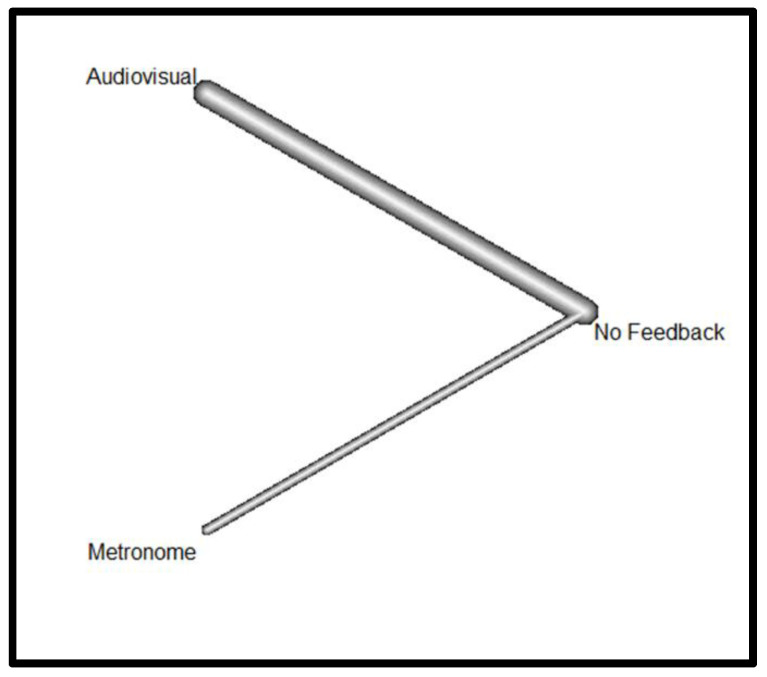
Netgraph for survival.

Figure 4 presents the Netgraph showing the different types of feedback devices (FDs) compared in terms of survival.

The Netgraph shows that there is a considerable comparison between audiovisuals and the absence of feedback devices (thick line), little comparison between the metronome and the absence of feedback devices (thin line), and no direct comparison between metronome and audiovisuals (absence of line). Therefore, the comparison between metronome and audiovisual devices is made indirectly through the feedback device.

Table 4 summarizes the results of the comparisons between different interventions within their respective studies.

The analysis of the overall effect relative to the applied interventions on survival shows OR 1.52 (IC 95% 1.27–1.81, *p* < 0.0001) for the audiovisual feedback and an OR of 1.19 (IC 95% 00.28–5.02, *p* = 0.81) for the metronome compared to patients managed without feedback.

**Figure 5:**
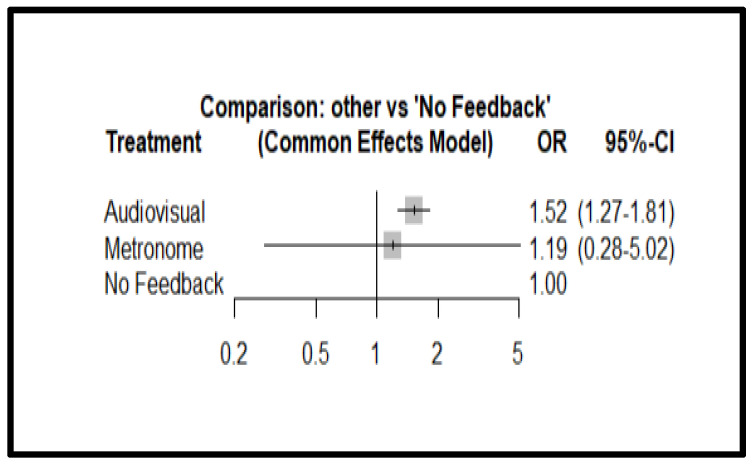
Forest plot for survival: Tau = 0.63, Tau^2^ = 0.40, I^2^ = 87.1% (74.3–93.5%).

Figure 5 shows the forest plot for ROSC, while the funnel plot with the distribution of effects concerning the standard error can be found in Supplementary Materials in Figure S2, Funnel plot for survival. 

### 3.3. Good Neurological Outcomes

**Figure 6:**
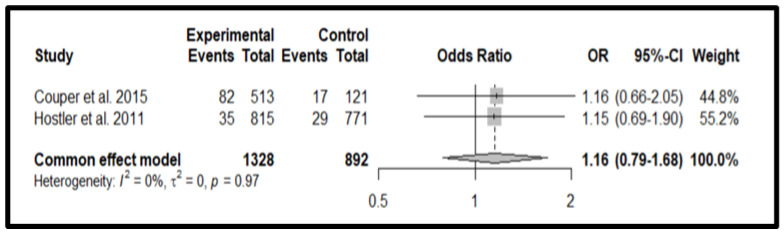
Forest plot for neurological status [15,26].

Figure 6 summarizes the results of the comparisons between audiovisual devices and unassisted CPR. The analysis of the overall effect relative to the applied interventions on survival shows OR 1.16 (95% CI 0.79–1.68, *p* = 0.45) for the audiovisual feedback.

The funnel plot with the distribution of effects concerning the standard error can be found in Supplementary Materials in Figure S3, Funnel plot for good neurological outcome. 

## 4. Discussion

The present meta-analysis, through direct and indirect comparisons, analyzed the impact of the use of feedback devices during cardiopulmonary resuscitation. In particular, the effects of audiovisual and metronome devices on ROSC, survival, and neurological outcomes were observed.

First, studies concerning the influence of audiovisual devices on the return to spontaneous circulation (ROSC) were analyzed. Analysis of the results regarding ROSC showed that compared to no devices in canonical CPR, the use of audiovisual feedback devices leads to an increase in ROSC (OR 1.26, 95% confidence interval [CI] 1.13–1.41%, *p* < 0.0001). In contrast, comparing classical CPR without device support to cardiopulmonary resuscitation supported by the use of metronomes, in the same way, showed that the latter did not lead to a significant increase in ROSC (OR 1.5, 95% confidence interval [CI] 0.98–2.23%, *p* = 0.06); however, it is possible that the use of the metronome was not significant due to a lack of studies or the inclusion of lower-level studies.

In the funnel plot we derived for ROSC, it could be observed that Egger’s statistical test did not show publication bias between studies that reported the incidence of ROSC in the use of the different feedback devices or their absence.

Secondly, the effects of audiovisual and metronome feedback devices on survival were observed. During the meta-analysis, several studies emerged comparing the use of audiovisual devices versus non-use and asking questions about their impact on survival. In contrast, there is a poor comparison in the literature of the impact of metronomes versus non-use concerning survival. Concerning the impact on survival, no direct comparison was found between the use of audiovisual feedback devices and metronomes, which could affect the results obtained. The meta-analysis showed that the support of audio-visual feedback devices has a positive impact on survival. The forest plot confidence interval indicates statistical significance (OR 1.52; 95% Confidence Interval [CI] 1.27–1.81).

In contrast, metronomes did not prove to have any impact on the outcome analyzed. This is justified by the very wide confidence interval, which highlights the statistical non-significance of their use (OR 1.19; 95% Confidence Interval [CI] 0.28–5.02). Thus, in contrast to ROSC, for which there are no significant data as there are statistically few studies using the metronome, it is likely that survival is not improved by the use of the metronome (*p*-value 0.81).

Finally, the effects of audiovisual feedback and metronomes on neurological outcomes were analyzed. In this regard, the literature presented a small number of studies analyzing the impact of devices on neurological outcomes. Specifically, these studies only observed the use of audiovisual devices and not metronomes. As a method of analysis, a non-network frequentist fixed-effect meta-analysis was performed. In this case, the effects are not distributed between the samples but there is only one true effect, and the differences between the studies are due to errors inherent in taking real-world measurements.

The meta-analysis showed that audio-visual device support did not significantly improve neurological status OR 1.16 (95% CI 0.79–1.68, *p* = 0.45) Looking at the forest plot in Figure 6, we can therefore state that the total effect is not significant as the tip of the cumulative effects diamond touches the baseline.

To assess the heterogeneity of studies within the meta-analysis, Kendall’s tau correlation coefficient, which quantifies the relationship between two variables, and the statistical I^2^, used to quantify heterogeneity between studies, were used. The I^2^ is based directly on the Cochran Q method and is defined as the percentage of variability in effect size that is not caused by sampling error.

In the cases of ROSC and survival, the percentages of I^2^ are in the range of 75% to 100%; more precisely, 80.3% for ROSC and 87.1% for survival. These percentages, according to the Cochrane Manual, are attributable to considerable heterogeneity. Concerning the result on neurological outcome, 0.0% indicates low heterogeneity.

It has been observed that in many cases, sub-optimal cardiopulmonary resuscitation is practiced both during resuscitation attempts in real cases and during simulated scenarios (guidelines for CPR and ECC, s.d.). Current guidelines consider correct cardiac massage, in terms of frequency, depth, and release, to be the principal standard for good CPR resulting in a return to spontaneous circulation and favorable outcomes [35]. To meet these standards, healthcare professionals need more and more training to maintain the ability to apply resuscitation skills. However, constant training may not be enough as the psychomotor skills of CPR delivery deteriorate rapidly [36,37].

A potential tool to ensure the maintenance of CPR skills, especially in those operators who practice basic life support infrequently, could be the use of stand-alone feedback devices or those connected to automated external defibrillators (AEDs) [38].

This meta-analysis showed that the support of feedback devices during cardiopulmonary resuscitation may lead to a positive impact on the return to spontaneous circulation and survival. In particular, audio-visual feedback devices may lead to better outcomes on the outcomes studied. However, studies supporting the use of metronomes are not numerous in the current literature.

Based on the available evidence, it can be affirmed that the use of audio-visual feedback devices should be encouraged both during training and during cases of cardiac arrest in real-life scenarios. These devices may prove to be a useful and supportive tool for the performer, especially if he/she does not work in emergency/urgent settings where it may be easier to maintain practical and theoretical skills over time.

The use of feedback devices would therefore ensure the better acquisition of skills during training and their maintenance during CPR in real time with a consequent improvement in patient outcomes. Furthermore, we must point out that the cost of the devices is not excessive and could be a useful tool to increase the survival of patients affected by IHCA by improving CPR.

We must underline that our analysis showed that there is a need for more studies on the OHCA setting, because pre-hospital systems, which often have to manage cardiac arrest in inaccessible and complicated situations, could benefit more from the use of these devices. In addition, studies should be set up to analyze the good neurological outcomes of patients, with a longer follow-up than simply ROSC; in fact, in the event of a return to spontaneous circulation, it does not guarantee that the patient will survive in the long term.

## 5. Conclusions

The meta-analysis showed that feedback devices are a valid support for increasing the probability of ROSC and survival for patients. Long-term survival data are lacking, however, so we would like to hope for increased interest in the scientific world. The effect of these devices is very efficient; thus, one could envisage their routine use within wards together with all the necessary devices for the management of IHCA.

## Figures and Tables

**Table 1:** Included studies.

Author	Year	Sample	Outcome	Feedback Type
Vahedian-Azimi et al. [25]	2016	80	-ROSC	Audiovisual
Couper et al. [26]	2015	634	-ROSC -SURVIVAL -NEUROLOGICAL OUTCOME	Audiovisual
Kramer-Johansen et al. [27]	2006	358	-ROSC -SURVIVAL	Audiovisual
Goharani et al. [28]	2019	900	-ROSC -SURVIVAL	Audiovisual
Obling et al. [29]	2022	325	-ROSC -SURVIVAL	Audiovisual, Metronome
Hostler et al. [15]	2011	1586	-ROSC -SURVIVAL -NEUROLOGICAL OUTCOME	Audiovisual
Lukas et al. [30]	2012	638	-ROSC	Audiovisual
Abella et al. [31]	2007	156	-ROSC -SURVIVAL	Audiovisual
Botelho et al. [32]	2016	111	-ROSC -SURVIVAL	Metronome
Camacho Leis et al. [33]	2012	892	-ROSC	Audiovisual
Lakomek et al. [16]	2020	292	-ROSC	Audiovisual
Agerskov et al. [34]	2017	196	-ROSC -SURVIVAL	Audiovisual

**Table 2:** Selected studies with the respective outcomes analyzed, type of intervention/control, number of events meeting the outcome per intervention/control, and the total number of events in the intervention/control group.

Study	Outcome	Feedback	Event	Total
Couper et al., 2015 [26]	GOOD NEUROLOGICAL OUTCOME	Audiovisual	82	513
Couper et al., 2015 [26]	GOOD NEUROLOGICAL OUTCOME	No Feedback	17	121
Hostler et al., 2011 [15]	GOOD NEUROLOGICAL OUTCOME	Audiovisual	35	815
Hostler et al., 2011 [15]	GOOD NEUROLOGICAL OUTCOME	No Feedback	29	771
Couper et al., 2015 [26]	SURVIVAL	No Feedback	21	121
Couper et al., 2015 [26]	SURVIVAL	Audiovisual	90	513
Kramer-Johansen et al., 2006 [27]	SURVIVAL	No Feedback	7	241
Kramer-Johansen et al., 2006 [27]	SURVIVAL	Audiovisual	5	117
Goharani et al., 2019 [28]	SURVIVAL	No Feedback	128	450
Goharani et al., 2019 [28]	SURVIVAL	Audiovisual	243	450
Hostler et al., 2011 [15]	SURVIVAL	No Feedback	96	771
Hostler et al., 2011 [15]	SURVIVAL	Audiovisual	92	815
Abella et al., 2007 [31]	SURVIVAL	No Feedback	5	55
Abella et al., 2007 [31]	SURVIVAL	Audiovisual	9	101
Botelho et al., 2016 [32]	SURVIVAL	No Feedback	4	60
Botelho et al., 2016 [32]	SURVIVAL	Metronome	4	51
Agerskov et al., 2017 [34]	SURVIVAL	No Feedback	53	134
Agerskov et al., 2017 [34]	SURVIVAL	Audiovisual	24	62
Vahedian-Azimi et al., 2016 [25]	ROSC	Audiovisual	29	40
Vahedian-Azimi et al., 2016 [25]	ROSC	No Feedback	14	40
Couper et al., 2015 [26]	ROSC	Audiovisual	262	513
Couper et al., 2015 [26]	ROSC	No Feedback	61	121
Kramer-Johansen et al., 2006 [27]	ROSC	Audiovisual	27	117
Kramer-Johansen et al., 2006 [27]	ROSC	No Feedback	42	241
Goharani et al., 2019 [28]	ROSC	Audiovisual	300	450
Goharani et al., 2019 [28]	ROSC	No Feedback	191	450
Obling et al., 2022 [29]	ROSC	Audiovisual	51	155
Obling et al., 2022 [29]	ROSC	Metronome	38	77
Obling et al., 2022 [29]	ROSC	No Feedback	38	93
Hostler et al., 2011 [15]	ROSC	Audiovisual	361	815
Hostler et al., 2011 [15]	ROSC	No Feedback	345	771
Lukas et al., 2012 [30]	ROSC	Audiovisual	165	319
Lukas et al., 2012 [30]	ROSC	No Feedback	151	319
Abella et al., 2007 [31]	ROSC	Audiovisual	45	101
Abella et al., 2007 [31]	ROSC	No Feedback	22	55
Botelho et al., 2016 [32]	ROSC	Metronome	28	51
Botelho et al., 2016 [32]	ROSC	No Feedback	36	60
Camacho Leis et al., 2012 [33]	ROSC	Audiovisual	50	104
Camacho Leis et al., 2012 [33]	ROSC	No Feedback	319	788
Lakomek et al., 2020 [16]	ROSC	Audiovisual	37	103
Lakomek et al., 2020 [16]	ROSC	No Feedback	69	189
Agerskov et al., 2017 [34]	ROSC	Audiovisual	34	62
Agerskov et al., 2017 [34]	ROSC	No Feedback	72	134

**Table 3:** Summary of effect sizes (common effect model).

Study	Treatment 1	Treatment 2	OR	95% CI	Leverage
Vahedian-Azimi et al., 2016 [25]	Audiovisual	No feedback	1.26	[1.13–1.41]	0.01
Couper et al., 2015 [26]	Audiovisual	No feedback	1.26	[1.13–1.41]	0.08
Kramer-Johansen et al., 2006 [27]	Audiovisual	No feedback	1.26	[1.13–1.41]	0.04
Goharani et al., 2019 [28]	Audiovisual	No feedback	1.26	[1.13–1.41]	0.17
Obling et al., 2022 [29]	Audiovisual	Metronome	0.84	[0.55–1.30]	0.00
Obling et al., 2022 [29]	Audiovisual	No feedback	1.26	[1.13–1.41]	0.00
Obling et al., 2022 [29]	Metronome	No feedback	1.50	[0.98–2.30]	0.00
Hostler et al., 2011 [15]	Audiovisual	No feedback	1.26	[1.13–1.41]	0.32
Lukas et al., 2012 [30]	Audiovisual	No feedback	1.26	[1.13–1.41]	0.13
Abella et al., 2007 [31]	Audiovisual	No feedback	1.26	[1.13–1.41]	0.03
Botelho et al., 2016 [32]	Metronome	No feedback	1.50	[0.98–2.30]	0.32
Camacho Leis et al., 2012 [33]	Audiovisual	No feedback	1.26	[1.13–1.41]	0.08
Lakomek et al., 2020 [16]	Audiovisual	No feedback	1.26	[1.13–1.41]	0.05
Agerskov et al., 2017 [34]	Audiovisual	No feedback	1.26	[1.13–1.41]	0.03

**Table 4:** Summary of effect sizes (common effects model).

Study	Treatment 1	Treatment 2	OR	95% CI	Leverage
Couper et al., 2015 [26]	Audiovisual	No feedback	1.52	[1.27–1.81]	0.12
Kramer-Johansen et al., 2006 [27]	Audiovisual	No feedback	1.52	[1.27–1.81]	0.02
Goharani et al., 2019 [28]	Audiovisual	No feedback	1.52	[1.27–1.81]	0.41
Hostler et al., 2011 [15]	Audiovisual	No feedback	1.52	[1.27–1.81]	0.34
Abella et al., 2007 [31]	Audiovisual	No feedback	1.52	[1.27–1.81]	0.02
Botelho et al., 2016 [32]	Metronome	No feedback	1.19	[0.28–5.02]	1.00
Agerskov et al., 2017 [34]	Audiovisual	No feedback	1.52	[1.27–1.81]	0.08

## Data Availability

The original data presented in the study are available in the manuscript.

## Supplementary Materials

The following supporting information can be downloaded at: https://www.mdpi.com/article/10.3390/jcm13195989/s1, Table S1: Research strategy; Figure S1: Funnel plot for ROSC; Figure S2: Funnel plot for survival; Figure S3: Funnel plot for good neurological outcome.
