# Supplementary material for: The Role of Different Feedback Devices in the Survival of Patients in Cardiac Arrest: Systematic Review with Meta-Analysis
Source: J Clin Med. 2024 Oct 8;13(19):5989. doi: 10.3390/jcm13195989 (PMC11477487; doi:10.3390/jcm13195989)

**Table S1.** Research strategy.

| **Tabella 1 – stringhe di ricerca specifiche per le diverse banche dati** | |
|---|---|
| **PUBMED** | ((cardiac arrest*[Title/Abstract]) OR (cardiopulmonary arrest*[Title/Abstract]) OR (heartarrest*[Title/Abstract]) OR (cardiopulmonary arrest[MeSH Terms]) OR (heart arrest[MeSHTerms])) **AND** ((real-time feedback[Title/Abstract]) OR (feedback device*[Title/Abstract]) OR(sensory device*[Title/Abstract]) OR (audiovisual device*[Title/Abstract]) OR(metronome*[Title/Abstract]) OR (audio-visual device*[Title/Abstract]) OR (visualdevice*[Title/Abstract]) OR (audio visual device*[Title/Abstract]) OR (feedbackprompt*[Title/Abstract]) OR (electronic device*[Title/Abstract]) OR (feedback*[Title/Abstract])) **AND** ((mortality[Title/Abstract]) OR (survival[Title/Abstract]) OR (outcome*[Title/Abstract])OR (mortality[MeSH Terms]) OR (analysis, survival[MeSH Terms]) OR (Return ofSpontaneous Circulation[MeSH Terms]) OR (Return of SpontaneousCirculation[Title/Abstract]) OR (ROSC[Title/Abstract])) |
| **EMBASE** | (("cardiac arrest*".tw.) OR ("cardiopulmonary arrest*".tw.) OR ("heart arrest*".tw.) OR (exp"cardiopulmonary arrest"/) OR (exp "heart arrest"/)) **AND** (("real-time feedback".tw.) OR("feedback device*".tw.) OR ("sensory device*".tw.) OR ("audiovisual device*".tw.) OR(metronome*.tw.) OR ("audio-visual device*".tw.) OR ("visual device*".tw.) OR ("audio visualdevice*".tw.) OR ("feedback prompt*".tw.) OR ("electronic device*".tw.) OR (feedback*.tw.)) **AND** ((mortality.tw.) OR (survival.tw.) OR (outcome*.tw.) OR (exp mortality/) OR (exp"analysis, survival"/) OR (exp "Return of |

| | |
|---|---|
| | Spontaneous Circulation"/) OR ("Return ofSpontaneous Circulation".tw.) OR (ROSC.tw.)) |
| **SCOPUS** | (("cardiac arrest*".tw.) OR ("cardiopulmonary arrest*".tw.) OR ("heart arrest*".tw.) OR (exp"cardiopulmonary arrest"/) OR (exp "heart arrest"/))<br>**AND**<br>(("real-time feedback".tw.) OR("feedback device*".tw.) OR ("sensory device*".tw.) OR ("audiovisual device*".tw.) OR(metronome*.tw.) OR ("audio-visual device*".tw.) OR ("visual device*".tw.) OR ("audio visualdevice*".tw.) OR ("feedback prompt*".tw.) OR ("electronic device*".tw.) OR (feedback*.tw.))<br>**AND**<br>((mortality.tw.) OR (survival.tw.) OR (outcome*.tw.) OR (exp mortality/) OR (exp"analysis, survival"/) OR (exp "Return of Spontaneous Circulation"/) OR ("Return ofSpontaneous Circulation".tw.) OR (ROSC.tw.)) |
| **WEB OF SCIENCE (WOS)** | (("cardiac arrest*".tw.) OR ("cardiopulmonary arrest*".tw.) OR ("heart arrest*".tw.) OR (exp"cardiopulmonary arrest"/) OR (exp "heart arrest"/))<br>**AND**<br>(("real-time feedback".tw.) OR("feedback device*".tw.) OR ("sensory device*".tw.) OR ("audiovisual device*".tw.) OR(metronome*.tw.) OR ("audio-visual device*".tw.) OR ("visual device*".tw.) OR ("audio visualdevice*".tw.) OR ("feedback prompt*".tw.) OR ("electronic device*".tw.) OR (feedback*.tw.))<br>**AND**<br>((mortality.tw.) OR (survival.tw.) OR (outcome*.tw.) OR (exp mortality/) OR (exp"analysis, survival"/) OR (exp "Return of Spontaneous Circulation"/) OR ("Return ofSpontaneous Circulation".tw.) OR (ROSC.tw.)) |

**Figure S1.** Funnel plot for ROSC.

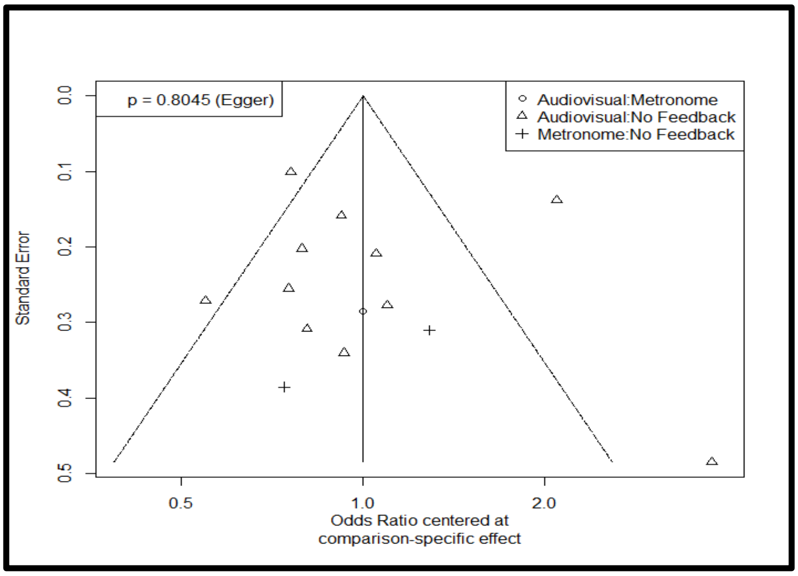


**Figure S2.** Funnel plot for survival.

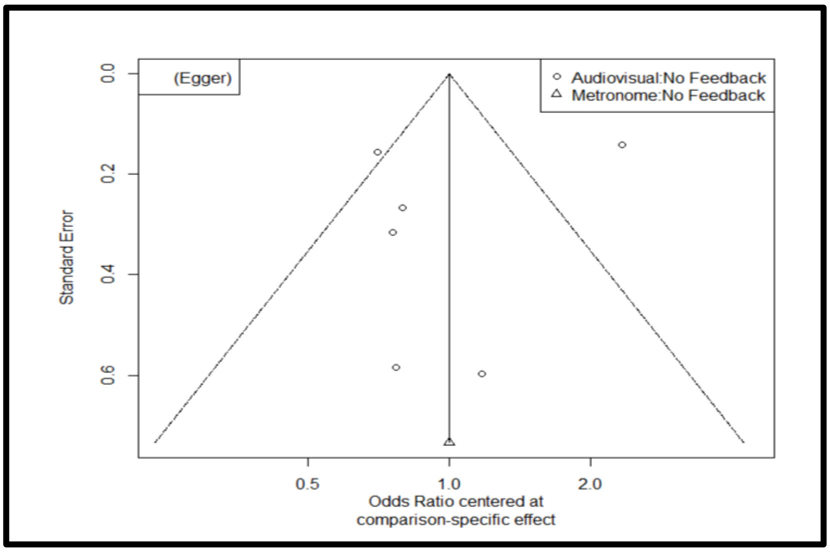

**Figure S3.** Funnel plot for good neurological outcome.

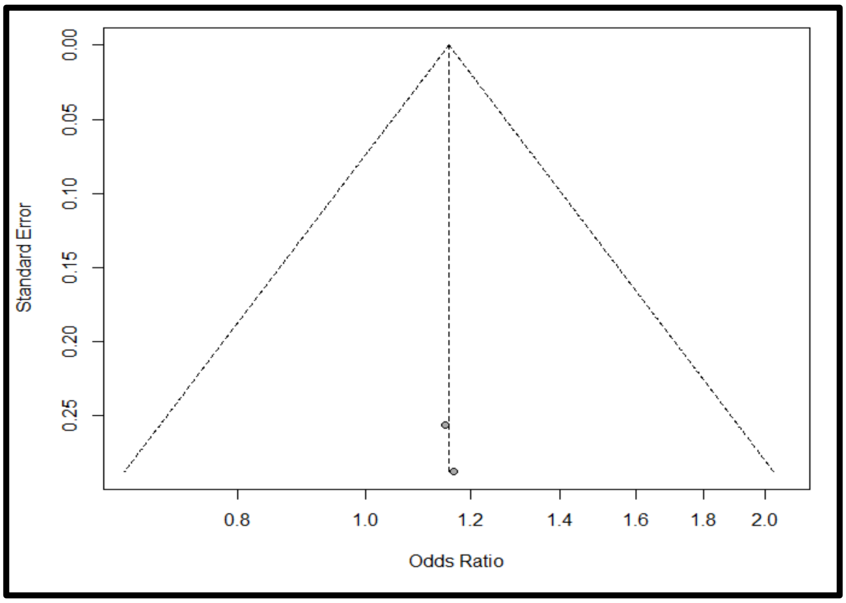

Supplement: Supplementary file 1 [file jcm-13-05989-s001.zip › jcm-3212786-supplementary.pdf]
